# Impact of (chemo)radiotherapy on immune cell composition and function in cervical cancer patients

**DOI:** 10.1080/2162402X.2016.1267095

**Published:** 2016-12-23

**Authors:** H. van Meir, R. A. Nout, M. J. P. Welters, N. M. Loof, M. L. de Kam, J. J. van Ham, S. Samuels, G. G. Kenter, A. F. Cohen, C. J. M. Melief, J. Burggraaf, M. I. E. van Poelgeest, S. H. van der Burg

**Affiliations:** a Department of Gynecology, Leiden University Medical Center, Leiden, the Netherlands; b Centre for Human Drug Research, Leiden, the Netherlands; c Department of Radiation Oncology, Leiden University Medical Center, Leiden, the Netherlands; d Department of Medical Oncology, Leiden University Medical Center, Leiden, the Netherlands; e Center Gynecological Oncology Amsterdam, NKI-AvL, Amsterdam, the Netherlands; f ISA Pharmaceuticals, Leiden, the Netherlands

**Keywords:** Cervical cancer, immunomonitoring, immunosuppression, PD-1, radiotherapy

## Abstract

New treatments based on combinations of standard therapeutic modalities and immunotherapy are of potential use, but require a profound understanding of immune modulatory properties of standard therapies. Here, the impact of standard (chemo)radiotherapy on the immune system of cervical cancer patients was evaluated. Thirty patients with cervical cancer were treated with external beam radiation therapy (EBRT), using conventional three-dimensional or intensity modulated radiation therapy without constraints for bone marrow sparing. Serial blood sampling for immunomonitoring was performed before, midway and at 3, 6 and 9 weeks after EBRT to analyze the composition of lymphocyte and myeloid-cell populations, the expression of co-stimulatory molecules, T-cell reactivity and antigen presenting cell (APC) function. Therapy significantly decreased the absolute numbers of circulating leukocytes and lymphocytes. Furthermore, the capacity of the remaining T cells to respond to antigenic or mitogenic stimulation was impaired. During treatment the frequency of both CD4^+^ and CD8^+^ T cells dropped and CD4^+^ T cells displayed an increased expression of programmed cell death-1 (PD-1). *In vitro* blocking of PD-1 successfully increased T-cell reactivity in all five samples isolated before radiotherapy but was less successful in restoring reactivity in samples isolated at later time points. Moreover, (chemo)radiotherapy was associated with an increase in both circulating monocytes and myeloid-derived suppressor cells (MDSCs) and an impaired capacity of APCs to stimulate allogeneic T cells. T-cell reactivity was slowly restored at 6–9 weeks after cessation of therapy. We conclude that conventional (chemo)radiotherapy profoundly suppresses the immune system in cervical cancer patients, and may restrict its combination with immunotherapy.

## Introduction

Radiotherapy is used as primary or adjuvant therapy in the curative treatment of patients with cervical cancer. Primary (chemo)radiotherapy is an effective treatment for locally advanced cervical cancer with a 5-y pelvic control rate of 87% and a cancer-specific survival of 79%.^1^ However, especially in cases with tumor cell positive lymph nodes and patients with higher stages, systemic failure of current therapies represents a major challenge. Radiotherapy was thought to mediate its effect through direct cytotoxic or cytostatic effects on malignant cells, but (pre)clinical findings suggest that its therapeutic effect also contains vascular and immunogenic components. Normalization of the vasculature by radiotherapy facilitates the delivery of chemotherapeutic compounds and promotes the infiltration by effector immune cells into the tumor bed.^2-4^ In addition, radiation therapy may alleviate immune suppression in the tumor microenvironment^5-7^ as well as (re)activate a tumor-specific cellular immune response.^6^ Changes in the tumor microenvironment contribute substantially to treatment success or failure, particularly in so-called immunogenic tumors.^8^ Interestingly, it was shown that a pre-treatment peripheral blood lymphocyte count at or above the median value was associated with higher clinical responses and survival rates. Apparently, peripheral blood lymphocyte count and lymphocyte subsets are independent predictors of survival and tumor regression in cervical cancer patients treated with concurrent chemoradiation.^9,10^

Cervical cancer is regarded as an immunogenic tumor since it is induced by a persistent infection with human papilloma virus (HPV), most often HPV16 or HPV18.^11^ The number and functional orientation of tumor-infiltrating CD4^+^ and CD8^+^ T cells and the presence of M1 type macrophages are strongly associated with survival in patients with cervical cancer after primary treatment.^12-15^ The upregulation of signaling through negative co-stimulatory molecules on T cells, such as Cytotoxic T-lymphocyte Antigen 4 (CTLA-4) and programmed cell death-1 (PD-1), is another mechanism through which T-cell infiltration and function can be impaired in cervical cancer.^16,17^ Whereas studies in mouse tumor models suggest that certain radiotherapy schedules can be combined with immunotherapy,^18-20^ the effect of standard radiation therapy in patients with cervical cancer has not been extensively studied. Most clinical studies investigated the baseline lymphocyte count as a prognostic predictor of treatment response,^9,10,21,22^ rather than the effects of therapy on the composition and function of these cells during treatment. Therefore, this study focused on the influence of pelvic radiation on immune responses in patients with cervical cancer during and after treatment. We prospectively analyzed changes in the immune cell composition and function during radiation therapy, with or without concomitant platinum-based chemotherapy, in serial blood samples from 30 patients with cervical cancer. We examined alterations in different lymphocytes subtypes, myeloid cell populations, the expression of co-stimulatory molecules, T-cell reactivity to antigens and the capacity of antigen presenting cells (APCs) to stimulate T cells. Our study showed that (chemo)radiotherapy for cervical cancer induced unfavorable immune changes reflected by a decreased number of circulating lymphocytes and an increased percentage in myeloid-cell populations, including myeloid-derived suppressor cells (MDSCs) and monocytes. Moreover, radiotherapy subverted the reactivity of T cells to antigenic stimulation and the capacity of APCs to enhance allogeneic T-cell proliferation. We further demonstrated that radiotherapy upregulates PD-1 expression on the circulating CD4^+^ T cells, which can partially explain their lower reactivity to antigenic stimulation.

## Results

### Characteristics of the patients

**Figure 1.**
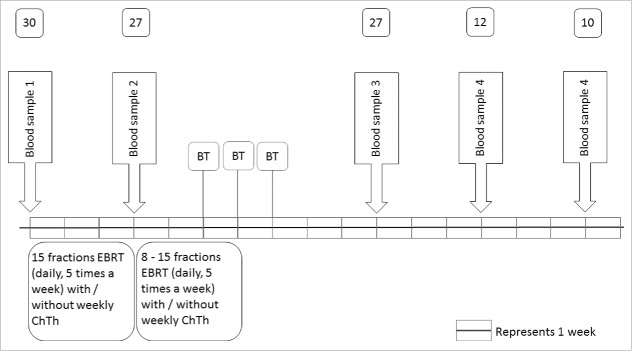
Treatment and blood sampling schedule. Blood samples 1–5: blood samples for immunomonitoring. Numbers above the blood samples indicate the amount of patients who provided blood for immunomonitoring. Abbreviations: EBRT = external beam radiation therapy; ChTh = chemotherapy; BT = brachytherapy.

Thirty patients with histologically proven invasive cervical cancer FIGO stage IB1 to IV, who were to receive EBRT with a curative intention, were enrolled between October 2011 and December 2014. A detailed treatment schedule is shown in Fig. 1. Patient characteristics are summarized in Table 1. Twenty-three patients (76.6%) received combined radiotherapy and chemotherapy, of which 18 with brachytherapy and 5 without. Four patients (13.3%) received radiotherapy alone, two patients (6.7%) received radiotherapy with brachytherapy and 1 patient (3.3%) received radiotherapy with hyperthermia. The patient receiving hyperthermia was initially scheduled for radiotherapy in combination with chemotherapy, but chemotherapy was canceled due to pre-existent renal impairment. Table S1 shows detailed information about all participating patients including treatment indication, dosages of the different treatment regimens and existence of high or low tumor load (high in case of primary or recurrence therapy; low in case of adjuvant, post-operative therapy). Three patients refused further participation after the first blood sampling and were considered as lost to immunological follow-up.

**Table 1.** Clinical and tumor characteristics of patients.

*N* = 30		
Number (percentage)		
Age (average)	48.9	(range 19–82)
FIGO stage		
IB1	11	(36.7%)
IB2	3	(10%)
IIA	1	(3.3%)
IIB	12	(40%)
IIIB	2	(6.7%)
IV	1	(3.3%)
Tumor size (average)	32.8 mm	(range 10–75 mm)
Treatment with		
EBRT + ChTh + BT	18	(60%)
EBRT + ChTh	5	(16.7%)
EBRT	4	(13.3%)
EBRT + BT	2	(6.7%)
EBRT + HT	1	(3.3%)
Treatment for		
Primary disease	18	(60%)
Adjuvant	8	(26.7%)
Recurrent disease	4	(13.3%)

For patients suffering from locally advanced cervical cancer, tumor size was measured before primary radiation therapy by magnetic resonance imaging (MRI). Age of the patients is given in years. Abbreviations: BT, brachytherapy; ChTh, chemotherapy; EBRT, external beam radiation therapy, FIGO = International Federation of Gynecology and Obstetrics, HT = hyperthermia.

### Radiation therapy has a profound effect on circulating immune cells

**Figure 2.**
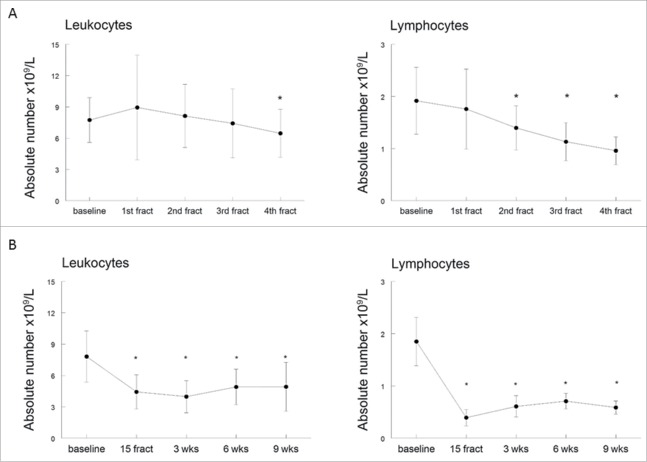
(Chemo)radiotherapy induced reduction in the absolute numbers of leukocytes and lymphocytes. (A) Time course of changes in the absolute number of leukocytes and lymphocytes before (baseline) treatment and during the first 5 d of fractionized radiotherapy. (B) Time course of changes in absolute number of leukocytes and lymphocytes before (baseline), during (15 fractions) and after (chemo)radiotherapy (3, 6 and 9 weeks after completion). Data are expressed as means ± SD, *p < 0.05. Abbreviations: fract = fractions; wks = weeks.

The routine leukocyte differential count obtained daily during the first week of EBRT, after 15 fractions of EBRT, and 3 weeks after completion of EBRT in the first 18 patients showed a decreased number of leucocytes in blood, which included a decrease in the absolute number of lymphocytes. This decrease in lymphocytes occurred early during treatment; already within 48 h after the first fraction a decrease of 27% was observed (from 1.916 × 10^9^/L (mean, range 0.96–3.37) at baseline to 1.397 × 10^9^/L (mean, range 0.5–1.99) after the second fraction; *p* = 0.0004) (Fig. 2A), and lasted at least until 3 weeks after completion of (chemo)radiation therapy (Fig. 2B). It was therefore decided to extend the observation period. An extended follow up of 6 weeks after completion of EBRT was done in 12 patients, and follow-up of 9 weeks after completion of EBRT was possible in 10 patients. All 30 patients showed the most distinct decrease of peripheral lymphocytes halfway the radiation treatment (after 15 fractions) with a mean absolute lymphocyte count of 0.39 × 10^9^/L, and showed a slight increase of lymphocyte count when radiation treatment was finished (Fig. 2B). Although at 6 weeks after completion of radiotherapy, the lymphocyte count was slightly increased compared to mid-treatment, it was still significantly lower when compared to baseline (*p* < 0.0001). This decrease in lymphocytes occurred regardless of tumor-load and of concurrent cisplatin treatment (Fig. S1A and B). These results suggest that (chemo)radiotherapy as used in cervical cancer patients is immunosuppressive.

### Radiotherapy reduces T-cell reactivity against common recall antigens and mitogens

**Figure 3.**
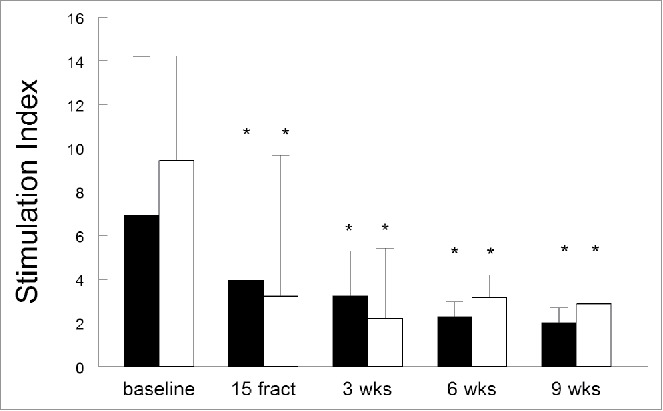
The effect of (chemo)radiotherapy on T-cell reactivity. The response of circulating T cells against memory response mix (MRM; black bars) and influenza M1 protein-derived peptides (FLU; white bars) was measured in the lymphocyte stimulation test (LST). T cell proliferation is expressed as stimulation index + standard error of the mean (SEM) and shown at different time points, including baseline, after 15 fractions of EBRT and 3, 6 and 9 weeks after completion of EBRT. Data were analyzed by mixed model and expressed as means + SEM. *p <0.05 with respect to baseline. Abbreviations: wks = weeks; fract = fractions.

PBMCs were stimulated with a pool of Influenza M1 peptides (FLU) and with a mix of bacterial recall antigens (MRM) to test if radiation therapy suppresses the capacity of T cells to respond to antigenic stimulation. In 29/30 patients (96.7%) the T-cell reactivity to FLU and MRM was strongly decreased during and shortly after completion of EBRT when compared to baseline (Fig. 3). Strikingly, the T-cell reactivity to MRM and FLU remained suppressed at 6 weeks and 9 weeks after completion of EBRT. The combined data show a severe decrease in the capacity of T cells to respond to stimulation with MRM and FLU after 15 fractions of EBRT (*p* = 0.0027 for MRM and *p* < 0.0001 for FLU). This situation continued until 6 weeks after termination of treatment (*p* = 0.0001 for MRM and *p* < 0.0001 for FLU) and was still retained at 9 weeks post-treatment, with mean stimulation index of 3.71 (*p* = 0.0091; Fig. 3). A sub-analysis comparing patients receiving either EBRT only or the combination of EBRT with cisplatin, showed a decrease in T-cell reactivity against both MRM and FLU regardless of the type of treatment (Fig. S1C and D). Interestingly, patients receiving EBRT only displayed higher baseline reactivity than those receiving chemoradiotherapy which nonetheless collapsed demonstrating the profound impact of EBRT on T-cell responsiveness.

Upon analysis of individual patient data it was noticed that four patients (ID3, ID5, ID8 and ID13) showed a high baseline T-cell response, a less severe decrease in T-cell reactivity and a persistent positive response during radiation therapy (ID3, ID8) or chemoradiotherapy (ID5, ID13) (Fig. S2A). Furthermore, in six (ID16, ID17, ID20, ID21, ID27, ID29, all treated with chemoradiation) of the 12 patients (50%) with the extended follow-up, a restoration of the proliferative T-cell response against FLU was noted at 6 and/or 9 weeks post-chemoradiation (Fig. S2B). These data showed that EBRT is associated with a loss in T-cell reactivity against common recall antigens, slowly recovering after cessation of therapy.

To gain more insight in the dynamics of T-cell reactivity during and after (chemo)radiotherapy, we decided to study the immune-cell composition and function of these 10 patients in more detail. First, the T-cell responses to PHA stimulation was studied and found to be strong for all 10 patients at baseline, with a mean stimulation index of 200.6 (range 54.0–586.0). The four patients who retained T-cell reactivity against the recall antigen FLU also showed a strong T-cell response to PHA stimulation throughout radiotherapy (mean stimulation index after 15 fractions was 325.75, range 121.0–446.0). For the six patients showing a chemoradiotherapy-induced reduction in recall antigen responsiveness, T-cell reactivity to PHA also significantly (*p* = 0.03) decreased to a mean stimulation index of 67.6 (range 15.0–123.0), albeit that reactivity was never completely lost (data not shown).

### Radiotherapy impairs the T-cell stimulatory capacity of APC

**Figure 4.**
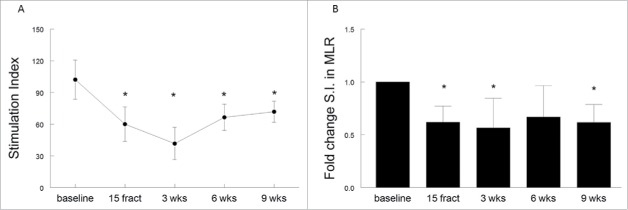
(Chemo)radiotherapy impairs the ability of antigen presenting cells to stimulate allogeneic T cells. Antigen presenting capacity of PBMCs as determined in a mixed lymphocyte reaction (MLR) in the blood samples from 10 patients are plotted over time. Treatment-induced changes in lymphocyte reactions as observed in the MLR based on (A) stimulation index (S.I.), expressed as mean ± SEM and (B) expressed as the fold changes (mean + SEM) of these S.I. over baseline. Time point include baseline, after 15 fractions of EBRT and at 3, 6 and 9 weeks after completion of EBRT. *p <0.05 with respect to baseline. Abbreviations: wks = weeks; fract = fractions.

The antigen presenting capacity of the patient's PBMCs was determined in a MLR.^23^ At baseline, the capacity of APCs to stimulate allogeneic T cells to proliferate was strong for all patients but a significant decrease in APC capacity was observed upon treatment (Fig. 4), with a mean fold change in stimulation index of 0.62 after 15 fractions, 0.56 at 3 weeks, 0.66 at 6 weeks and 0.62 at 9 weeks after treatment compared to baseline. Two of the patients were treated with EBRT only, and the eight other patients concurrently received cisplatin. Nonetheless, the suppressive treatment effect on APC capacity occurred regardless the therapy. Note, however, that the group of EBRT only is very small. Altogether, (chemo)radiation not only altered the number of circulating lymphocytes but also impaired the capacity of circulating APC to stimulate allogeneic T-cell proliferation in mixed lymphocyte reactions, shown as the decreased T-cell responsiveness within patient's PBMC to recall antigens and mitogens.

### Suppressive myeloid cell populations are more radioresistant than lymphocytes

**Figure 5.**
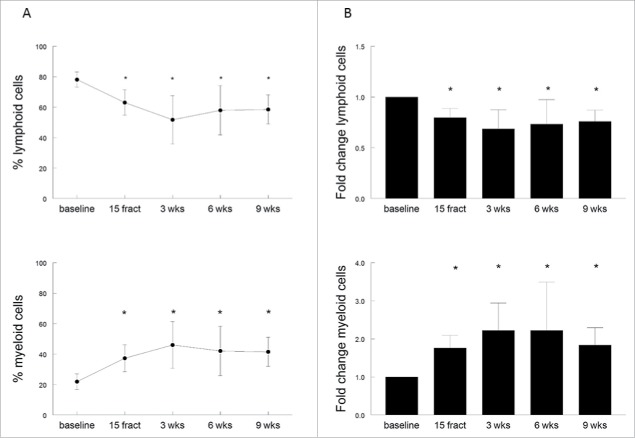
(Chemo)radiotherapy alters the relative frequencies of circulating myeloid and lymphoid cells. (A) Percentages of lymphoid cells (CD3^+^CD19^−^) and myeloid cells (CD3^−^CD19^−^) of viable cells as measured by flow cytometry. Mean percentages are shown for 10 patients with a complete follow-up at different time points, including baseline, after 15 fractions of EBRT and at 3, 6 or 9 weeks after completion of EBRT. Percentage are expressed as mean ± SD. (B) Fold changes in lymphoid and myeloid cells over baseline. Fold changes are expressed as mean + SEM; *p <0.05 with respect to baseline. Abbreviations: wks = weeks; fract = fractions.

The loss of T-cell reactivity and stimulatory capacity of APC in these 10 patients was associated with changes in the immune cell composition. After 15 fractionated doses of radiation therapy a significant decrease in the percentage of circulating lymphoid (CD3^+^CD19^−^) cells, and a concomitant increase in myeloid (CD3^−^CD19^−^) cell populations occurred (*p* = 0.0002 and *p* = 0.0006, respectively). This effect on circulating lymphoid cells (*p* < 0.0001) and myeloid cells (*p* < 0.0001) was still present after completion of the therapy, and was most prominent at 3 weeks after last EBRT (Fig. 5). Six to nine weeks after completion of radiotherapy, a slight increase of lymphoid cells and decrease of myeloid cells was observed, but the percentages remained significantly changed when compared to baseline. The effect on circulating lymphoid and myeloid cells occurred regardless the administration of cisplatin since similar kinetics in the percentages of lymphoid cells and myeloid cells are seen in patients treated with EBRT only (two patients) and the patients with cisplatin + EBRT (eight patients; Fig. S3A and B). A more in-depth analysis of the myeloid cell populations was based on of the expression of HLA-DR, to distinguish between macrophages or dendritic cells (DCs) (both HLA-DR^+^) and MDSC (HLA-DR^−/low^). In addition, the differential expression of CD14 and CD11b within the HLA-DR^+^ myeloid cell population was used to identify five previously reported subpopulations.^23^ For all the 10 patients a significant (*p* = 0.009) increase in the percentage of CD3^−^CD19^−^CD1a^−^HLA-DR^+^ myeloid cells was observed after 15 fractions of radiotherapy. This effect was stronger at 3 weeks (*p* < 0.0001), and the percentage of circulating myeloid cells remained significantly elevated at 9 weeks after completion of the radiotherapy (*p* = 0.0005), when compared to baseline (Fig. S4A). This effect was most pronounced for the population of CD14^+^CD11b^+^ expressing monocytes (Fig. S4B; *p* = 0.0038) and was retained after completion of the treatment. Although the percentage of circulating CD3^−^CD19^−^CD1a^−^HLA-DR^−^CD14^+^CD15^−^ monocytic MDSC (mMDSC) were very low at baseline (average 0.11%, range 0.02–0.31%), they increased in 9 out of the 10 patients upon radiotherapy, with a mean percentage in mMDSCs of 0.28% (range 0.04–1.32%; data not shown). Interestingly, the loss of T-cell reactivity against the recall antigen FLU was paralleled by the decrease in lymphoid cells and an increase in subpopulations of less stimulatory or even suppressive types of myeloid cells (Fig. S5). These data show that some myeloid cell populations are more radioresistant than lymphocytes and that their presence is associated an impaired T-cell response to recall antigens as well as an impaired antigen presenting cell capacity to stimulate allogeneic T cells. This suggests an overall immunosuppressive effect of (chemo)radiotherapy on systemic immunity in patients with cervical cancer.

### A specific increase in PD-1 expression by T cells upon radiotherapy

**Figure 6.**
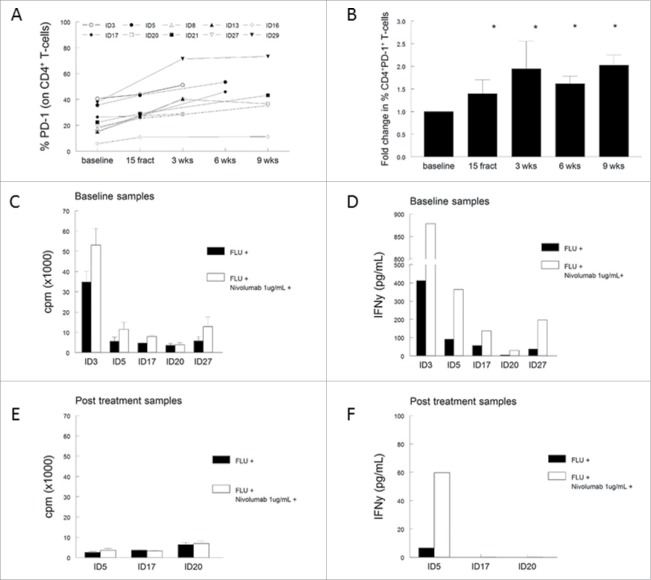
(Chemo)radiotherapy induces CD4^+^ T-cell suppression via PD-1. (A) Percentage of PD-1^+^ expressing CD4^+^ T cells for individual patients. (B) Aggregated fold changes of percentages with respect to baseline, *p <0.05 with respect to baseline. (C, D) Stimulation of five baseline PBMC samples with influenza Matrix 1 protein-derived peptides (FLU) *in vitro* in the presence (white bars) or absence (black bars) of PD-1 blocking using 1 μg/mL Nivolumab. (E, F) Stimulation of three post-treatment samples with FLU *in vitro* in the presence (white bars) or absence (black bars) of PD-1 blocking with 1 μg/mL Nivolumab. Displayed in (C) and (E) is the proliferation, expressed as counts per minute (cpm), shown as mean of triplicate wells plus standard deviation after stimulation. (D, F) Cytokine IFNγ production as measured within the supernatant of the proliferation assay, with and without PD-1 blocking. Abbreviations: wks = weeks; fract = fractions; cpm = counts per minute.

Analysis of the T-cell populations in PBMCs showed a strong reduction in the percentage of both CD4^+^ and CD8^+^ T cells in 9 of the 10 analyzed patients. However, the percentage of CD4^+^CD25^+^CD127^−^Foxp3^+^ regulatory T cells (Treg) and CD4^+^CD25^+^CD127^−^Foxp3^+^CD45RA^−^ activated regulatory T cells (aTreg) were not significantly altered during treatment (Fig. S6). We previously demonstrated that the frequency of CD4^+^ and CD8^+^ T cells expressing the co-inhibitory marker program death-1 (PD-1) was increased in cervical cancer patients when compared to healthy controls.^23^ Therefore, the expression of PD-1, cytotoxic T-lymphocyte antigen-4 (CTLA-4) and T-cell immunoglobulin mucin-3 (TIM-3) was analyzed to determine whether radiotherapy influences the expression of these inhibitory markers. Although the percentage of CTLA-4 and/or TIM-3 expressing CD4^+^ or CD8^+^ T cells remained similar upon treatment, we observed a high expression of PD-1 on a minority of circulating CD4^+^ T cells at baseline (range 5.8–40.6%), which increased up to 2.7-fold upon radiotherapy (range 10.8–71.4%). These higher percentages of PD-1 expressing CD4^+^ T cells occurred in all patients (regardless whether EBRT was combined with concurrent cisplatin (eight patients) or not (two patients; Fig. S7) and remained elevated for up to 9 weeks after radiotherapy (Fig. 6A and B). A similar effect was seen on circulating CD8^+^ T cells, with a significant increase of PD-1 expression 3 weeks after (chemo)radiotherapy when compared to baseline (*p* = 0.002, data not shown). The increase of PD-1 expression of CD4^+^ T cells upon treatment was accompanied with the decline in T-cell response against viral (FLU) antigens (Fig. S7). Notably, compared to the other nine patients, patient ID29 displayed a remarkably high level of CD4^+^-PD1^+^ T cells at baseline (39%) and concurrently the lowest T-cell reactivity in the LST, suggesting that PD-1 expression may have contributed to the insufficient immune response upon treatment with chemoradiotherapy. We subsequently explored whether blocking of the PD-1 signaling could improve T-cell responses. We used the PD-1 blocking antibody Nivolumab and stimulated five baseline and three post-chemoradiotherapy PBMC samples, which were still available to us, with autologous monocytes pulsed with a pool of FLU peptides. As a control, non-blocked PBMCs were used. We observed higher antigen-specific T-cell proliferation in four and increased IFNγ production in five out of the five tested baseline PBMC samples when Nivolumab was present (Fig. 6C and D), indicating that PD-1 expression contributed to the immune suppression in patients with cervical cancer. Unfortunately, this effect of anti-PD-1 was seen in only one out of the three post-chemoradiotherapy samples; the antigen-specific IFNγ production was partly restored for patient ID5 (Fig. 6E and F). Apparently, radiotherapy disturbs immunity via multiple other pathways, rendering concurrent PD-1 blocking less adequate for restoration of T-cell reactivity against recall antigens in this setting.

## Discussion

In this study, the immunological effects of standard (chemo)radiotherapy in patients with cervical cancer were studied. Radiotherapy without bone marrow sparing induced a substantial and long-lasting immune suppression. From the first fractions onwards, a decrease in the number of circulating lymphocytes, a decrease in T-cell reactivity to common recall antigens and a decrease in the capacity of APC to stimulate T-cell responses were found. Immune reactivity slowly recovered after cessation of therapy with only half of the patients responding 9 weeks after therapy. These effects on peripheral immune cells, T-cell function and APC capacity were similar for patients treated with radiation therapy alone or with concomitant chemotherapy, confirming an earlier report showing that radiotherapy alone and radiotherapy with concomitant cisplatin decreased the absolute number of all lymphocyte subsets and decreased PHA-induced T-cell proliferation.^24^

Chemoradiotherapy was also reported to be immune suppressive in patients with HPV-related oropharyngeal cancer.^25^ A decrease in CD4^+^ and CD8^+^ T cells, an increase of MDSCs and an unfavorable CD8^+^/Treg ratio upon radiation treatment was seen. Furthermore, an upregulation of PD-1 expression on CD4^+^ T cells was noted, which occurred 3 weeks after completion of therapy and remained elevated for up to 1 y following therapy.^25^ Although the radiation field in this patient group contains less active bone marrow, the immunosuppressive effects were long lasting (up to 1 y after treatment), indicating the direct effect of radiotherapy on the peripheral blood cells. PD-1 is a key immune checkpoint protein expressed on activated and exhausted T cells, which leads to the suppression of T-cell activity through interaction with its ligand PD-L1. We also observed elevated PD-1 expression on CD4^+^ T cells during and following radiotherapy and showed that this was associated with the impaired T-cell reactivity against FLU and impaired ability of APCs to stimulate allogeneic T cells. Together, this is strongly suggestive for radiotherapy induced immune suppression. There are a number of clinically available antibodies to block PD-1 signaling (e.g., Nivolumab, Pembrolizumab and Lambrolizumab). We hypothesized that PD-1 blockade could reverse radiotherapy-induced immune suppression. While our *in vitro* experiments in blocking PD-1 in PBMC samples isolated before radiotherapy provide further support for targeting PD-1 and its ligand in cervical cancer, this was hardly the case after the initiation of radiotherapy. PD-1 blocking in patients treated with (chemo)radiotherapy partly restored cytokine production but not proliferation of antigen-stimulated T cells. These data do not support a combination of the (chemo)radiotherapy together with PD-1/PD-L1 blockade to obtain more clinical benefit.

An increase in circulating MDSCs and myeloid cells upon (chemo)radiotherapy and a change in capacity of circulating APCs, reflected in a subverted reactivity of T cells to recall antigen stimulation as well as a lower response of allogeneic T cells to become activated was observed after completion of radiotherapy. This suggests that the loss of T-cell reactivity and stimulatory capacity of APC may also be caused by changes in the immune cell composition, in favor of (suppressive) myeloid cell populations.

Our results indicate that it will be a considerable challenge to establish the optimal delivery and dosing strategies when combining radiotherapy and immunotherapy before combinations hereof can successfully be applied to cervical cancer patients. In pre-clinical models, it was demonstrated that α radiation-based therapy (an *in situ* ablation treatment based on intra-tumoral^224^Ra-loaded wires that release its daughter atoms) inhibited breast, colon and lung tumor growth by stimulating anti-tumor immunity. It was suggested that combinations of local ablation treatments and immunotherapy could further augment powerful anti-tumor immunity.^26^ However, translation of radio-immunotherapy to the clinic requires careful consideration of the radiation dose and fractionation for both the tumor and organs at risk, particularly the bone marrow. Hematologic toxicity, including lymphopenia, is frequently noted in women undergoing pelvic radiotherapy for cervical cancer, because approximately 40% of the active bone marrow is located in the pelvic region, and T cells constantly circulate though this irradiation field.^27,28^ The extreme sensitivity of active pelvic bone marrow was recently demonstrated by McGuire et al. using [^18^F]Fluorothymidine (FLT) imaging using positron emission tomography (FLT-PET) to identify active bone marrow before, during and after radiotherapy.^29^ As little as a radiation dose of 4–5 Gy resulted in an approximately 50% decrease in FLT uptake and the suppression of bone marrow activity was measurable up to 1 y after radiotherapy, especially in pelvic cancer patients receiving radiation doses of more than 35 Gy.^29^ Based on empirical experience, the use of daily fractions of 2 Gy to a total dose of approximately 46–50 Gy has evolved as a standard radiotherapy approach to control microscopic disease for most tumor types including cervical cancer. It is possible that different treatment regimens, depending on fractioning, dosing and delivery may have different effects on antitumor immunity. In the setting of cervical cancer, an immune enhancing effect was seen in the tumor-draining lymph nodes of patients undergoing low-dose radiation (total dose 39.6 Gy), while an immunosuppressive effect was observed in patients treated with high-dose radiation (total dose 50 Gy). Although dose differences were only minor, lower-dose radiation was associated with an increase in the anti-tumor Th1 and cytotoxic T-cell subsets, while a lower frequency of Tregs was noticed when compared to higher-dose radiation therapy.^30^ Furthermore, local low-dose gamma irradiation (2 Gy) caused normalization of aberrant vasculature and increased recruitment of tumor-specific T cells into human pancreatic tumors, by the polarization of M2-like toward M1-like macrophages.^31^

This underlines again the importance of further characterization of the effect of radiotherapy on the immune system. To reduce the incidence and severity of hematologic toxicity, the use of techniques that limit pelvic bone marrow irradiation is of interest.^32,33^ Especially bone marrow sparing (BMS) intensity modulated radiotherapy (IMRT) is a technique that could reduce the volume of bone marrow receiving high dosages, while maintaining target coverage, resulting in less hematologic toxicity,^29^ and potentially limit the suppressive effect on lymphoid populations and immune responses. Until now, there is no clear evidence for an optimal radiation dose fractionation schedule based on clinical data to elicit antitumor immune responses, and there is a lack of (randomized) studies comparing radiation regimens (with or without bone marrow sparing) for their ability to synergize with immunotherapy. The optimal dose and fractionation schedule should cause sufficient cytotoxic effects for tumor eradication, while reducing myeloid cell associated suppressive effects and foster lymphoid cell populations and effective immune responses. As peripheral blood lymphocyte count and lymphocyte subsets have shown to be independent predictors of survival and tumor regression in cervical cancer patients treated with concurrent chemoradiation,^9,10^ such immunological markers could be used to select the optimal (combination) treatment schedule for the patient that benefits most.

There were some limitations of this study. Our analyses were limited to the systemic immunity, rather than direct examination of the intra-tumoral cell composition itself. It remains to be established whether (chemo)radiotherapy-induced alterations in circulating immune cells also occurs at the tumor site. It has been shown in head and neck cancer patients that tumor infiltrating T cells have a higher expression of PD-1 compared to circulating T cells.^34^ In addition, cryopreservation may cause downregulation of PD-1 and PD-L1 expression on PBMCs. This implies that our findings on radiotherapy-induced increased PD-1 expression on CD4^+^ T cells may underestimate the effect of radiotherapy on the tumor and its microenvironment. Another limitation is the relatively small number of patients participating in this trial (*N* = 30) and the variation in the amount of blood samples provided by patients. Due to clinical conditions and disease burden, motivating patients to donate blood was difficult. A further limitation is the heterogeneity in the participating patients. Differences in FIGO stage, treatment (adjuvant versus primary) and clinical performance status, made it difficult to study potential interesting differences between patient groups. In addition, there was a high variation in treatment modalities. Although every patient was treated with high dose EBRT without bone marrow sparing, there were different additional treatment regimens including concurrent chemotherapy, hyperthermia and/or brachytherapy.

In conclusion, our data show that standard non-BMS radiotherapy affects circulating immune cells and immune responses, causing immune suppression in patients with cervical cancer. Relevant mechanisms underlying this (chemo)radiotherapy-induced immunosuppression include decrease in lymphoid cells, decrease of APC function, increase of different subsets of myeloid cells and PD-1 upregulation on T cells. Studies on the immunological effects of BMS radiotherapy should be made, in order to determine whether alternative treatments as immunotherapy could synergistically improve immune responses and outcomes.

## Materials and methods

### Ethics

The Medical Ethics Committees of the Leiden University Medical Center (LUMC) and the Netherlands Cancer Institute-Antoni van Leeuwenhoek hospital (NKI-AvL) formally approved the protocol of this study. The study was conducted according to the Dutch Act on Medical Research involving Human Subjects (WMO) and was registered under number NL36829.058.11. Written informed consent was obtained from all patients before study inclusion and participation. All participating patients were acknowledged that they were fully anonymized and cannot be identified via the paper.

### Patients and treatments

This observational study was performed at the gynecology clinics of the LUMC and the NKI-AvL from October 2011 to December 2014. Thirty patients with invasive cervical cancer (FIGO stage IB1 to IV^35^) with an indication for external beam radiation therapy (EBRT) were recruited for participation. Additionally, the eligibility of patients required all of the following criteria: mentally competent patients of 18 y and older, no other active malignancy than cervical cancer, no indication of active infectious disease such as HIV and hepatitis B, and no medical condition that may interfere with the study objectives.

Radiotherapy was used as primary treatment for locally advanced disease (FIGO stages IB2-IIIB, or lymph node positive), in combination with concurrent cisplatin chemotherapy and brachytherapy (BT). As adjuvant therapy after radical hysterectomy and pelvic lymphadenectomy for early stage disease, radiotherapy was applied in case of high-risk early stage disease with two or three unfavorable tumor characteristics.^36-39^ Unfavorable tumor characteristics included tumor diameter exceeding 40 mm, tumor depth more or equal to 15 mm and lymphovascular space involvement (LVSI). Patients with two or more tumor-positive lymph nodes, parametrial infiltration, or tumor-positive surgical margins were treated with radiotherapy and concurrent cisplatin chemotherapy. Patients suffering from recurrent cervical cancer with only surgical treatment in history were treated with EBRT in combination with chemotherapy, with or without BT. Patients with a contra-indication for cisplatin treatment were treated with 5 weekly courses of deep tissue hyperthermia.

All treatments were performed in accordance with the guidelines of the radiotherapy departments of the participating hospitals. EBRT was delivered in 23 fractions of 2 Gy (total 46 Gy) or an equivalent dose given in fractions of 1.8 Gy; 5 times a week. An EBRT boost was given in patients with PET-CT suspected lymph node metastases, aiming for a total dose of 60 Gy, taking the BT dose contribution into account. Standard EBRT was delivered using either conventional three-dimensional (3D-CRT) or intensity modulated radiation therapy (IMRT) without constraints for bone marrow sparing. Concurrent chemotherapy consisted of 5 or 6 weekly cycles of intravenous cisplatin (40 mg/m^2^ per cycle). Dose adjustments, omissions and delays were implemented as the standard intravenous cisplatin administration protocol of the institutes. Upon completion of EBRT, MRI-guided intracavitary alone or combined interstitial–intracavitary high dose rate BT was administered in three or four fractions of 7 Gy.^40^ The dose rate and fractionation was performed according to the department policy, aiming at an equivalent dose in 2 Gy fractions (EQD2 dose) of at least 80–85 Gy in high risk-clinical target volume (HR-CTV) according to the Groupe Européen de Curiethérapie (GEC) and the European Society for Radiotherapy & Oncology (ESTRO) guidelines.^41^ The aim was to maintain the overall treatment time within 7 weeks.

### Blood sampling and follow-up

Venous blood sampling for routine leukocyte differential count analysis took place daily during the first week of EBRT. These samples were analyzed for leukocyte differentiation at the routine laboratories of LUMC or NKI-AvL. Additionally, a full blood count and blood sampling for immunomonitoring were performed before start of radiotherapy (baseline), after 15 fractions of EBRT (midway) and at 3 weeks after completion of EBRT. Analysis of the data obtained in the first 15 patients at 3 weeks after therapy showed substantial decreases in lymphocyte counts and an impaired capacity of peripheral blood mononuclear cells (PBMCs) to respond to antigenic stimulation. Therefore, the protocol was amended to allow extra blood samples for immunological analysis at 6 and 9 weeks after completion of radiation therapy for the subsequent 15 patients. Twelve patients of the second group consented for these additional blood draws at 6 and 9 weeks. Fig. 1 shows treatment schedules in detail, with exact intervals between treatments and blood samples.

### Immunomonitoring

Venous blood samples for immunomonitoring were taken in 6 heparinized tubes of 9 mL to isolate PBMCs and in one 9 mL clot activator tube to obtain serum. Blood samples were transported at room temperature and PBMCs were isolated by Ficoll gradient centrifugation within 6 h. Part of these freshly obtained PBMCs was used for the lymphocyte stimulation test (LST). The remaining cells were cryopreserved in 90% fetal calf serum (PAA Laboratories, Pashing, Austria) and 10% DSMO at a concentration of 7 to 12 million cells per vial in a total volume of 1 mL using a mister Frosty's freezing container (Nalgene). Upon cryopreservation the vials were stored in the vapor phase of the liquid nitrogen until further use. Immunological assays were performed and analyzed under blinding for clinical parameters of the participating patients.

### T-cell proliferation assays

The proliferative response to the memory response mix (MRM) and influenza matrix 1 protein-derived peptides (FLU)^40^ was determined using freshly isolated PBMCs that were subjected to the LST as described previously.^23,42^ In short, eight replicate wells with 1.5×10^5^ cells per well were stimulated for 6 d with the indicated antigens (10 µg/mL), after which 50 μL supernatant per well was harvested, pooled for the eight similar wells and stored at −20°C for cytokine analysis. The cells were pulsed with 10 µCi/mL [^3^H]-thymidine (PerkinElmer, the Netherlands). The negative control consisted of cells in medium (Iscove's Modified Dulbecco's Medium, IMDM (Lonza) plus 10% human AB serum (Life Technologies)) only. A positive response was defined as a stimulation index (S.I.) of at least 3 under the condition that six out of eight wells displayed values above the cut off value, which was defined as the mean value of the cells in medium only plus 3× standard deviation (SD).

The capacity to respond to Phytohemagglutinin (PHA) was studied using cryopreserved PBMCs.^23^ Cells were thawed and tested in a 3-d proliferation assay with the minor alteration that 50,000 cells per well (in quadruplicate) were incubated in medium (IMDM) or stimulated with 0.25 µg/mL PHA (Murex Biotech HA16). At day 2, 100 µL supernatant per well was harvested for cytokine analysis, and cells were subjected to [^3^H]-thymidine (50 µL/well of 10 µCi/mL) for an additional 16–20 h. A positive response was defined as an S.I. of 3 or higher.

### Antigen presenting capacity assay

The antigen presenting capacity of the patient's PBMCs was determined in a mixed lymphocyte reaction (MLR).^23^ PBMCs were thawed in IMDM plus 10% fetal calf serum and 30 µg/mL DNase (Sigma-Aldrich, St. Louis, USA), pelleted and suspended in IMDM plus 10% human AB serum. Then, irradiated (3,000 rad) to prevent proliferation, washed, suspended in IMDM plus 10% human AB serum and plated at 1×10^5^ cells per well (in quadruplicate). Third party PBMCs of two donors were added to determine their proliferative capacity upon encountering the irradiated patients’ APCs. Third party PBMCs only as well as irradiated patients PBMCs only were used as negative controls. At day 6, 100 µL supernatant per well was harvested for cytokine analysis, and the cells were subjected to [^3^H]-thymidine (50 µL/well of 10 µCi/mL) for an additional 16–20 h. A positive response was defined as an S.I. of at least 3.

### Cytokine analysis

The supernatants harvested in the proliferation and MLR assays were subjected to a flow cytometer based cytokine bead array (CBA, human Th1/Th2 kit, BD), which was conducted according to the manufacturer's instructions and as reported earlier.^42^ The cytokine panel consisted of IFNγ, TNF-α, IL-10, IL-5, IL-4 and IL-2. A positive response was defined as a cytokine concentration above the detection limit as indicated by the manufacturer, which was 20 pg/mL for each cytokine. Treatment-related change in cytokine production was defined as a cytokine concentration above the cut-off value and a 3-fold increase or decrease above the baseline sample (pre-radiotherapeutic treatment).

### Phenotyping of PBMCs

The PBMC samples isolated at different time points were phenotyped as described earlier by using three sets of 10–13 cell surface markers to identify immune cell subsets and the expression of co-inhibitory molecules by flow cytometry.^23^

The myeloid set consists of CD3-HV450 (Clone UCHT1; BD Biosciences), CD1a-FITC (Clone HI149; BD Biosciences), CD11b-PE (Clone D12; BD Biosciences), CD11c-BV650 (Clone B-ly6; BD Biosciences), CD14-AF700 (Clone M5E2; BD Biosciences), CD15-PE-CF594 (Clone W6D3; BD Biosciences), CD19-BV605 (Clone SJ25C1; BD Biosciences), CD33-PE-Cy7 (Clone P67.6; BD Biosciences), CD56-PerCP-Cy5.5 (Clone HCD56; BioLegend), CD163-APC (Clone 215927; R&D Systems), CD206-APC-Cy7 (Clone 15–2; BioLegend) and HLA-DR-V500 (Clone L243; BD Biosciences).

The inhibitory set consists of CD3-HV450 (Clone UCHT1; BD Biosciences), and CD4^+^-PE-CF594 (Clone RPA-T4; BD Biosciences), CD8^+^-APC-Cy7 (Clone SK1; BD Biosciences), CD56-AF700 (Clone B159; BD Biosciences), CD94-FITC (Clone 131412; R&D Systems), CD152-PE-Cy5 (anti-CTLA-4, Clone BN13; BD Biosciences), CD279-PE-Cy7 (anti-PD-1, Clone EH12.2H7; BioLegend), TIM-3-BV605 (Clone F38.2E2; BioLegend) and CD159a-PE (NKG2a, Clone Z199; Beckman Coulter).

The regulatory T-cell set consists of CD3-HV500 (Clone UCHT1; BD Biosciences), and CD4^+^-AF700 (Clone RPA-T4; BD Biosciences), CD8^+^-PerCP-Cy5.5 (Clone SK1; BD Biosciences), CD25-PE-Cy7 (Clone 2A3; BD Biosciences), CD127-BV650 (Clone HIL-7R-M21; BD Biosciences), CD45RA-APC-H7 (Clone HI100; BD Biosciences), CD152-BV421 (CTLA-4, Clone BNI3, BD Biosciences); FoxP3-PE-CF594 (Clone 259D/C7; BD Biosciences), Helios-APC (Clone 22F6; BioLegend) and Ki67-FITC (Clone 20Raj1; eBioscience).^43^

The cryopreserved PBMCs were thawed and first subjected to live-dead marker (Yellow amino reactive dye (ARD); dilution 1:800) incubation for 20 min at room temperature in 100 µL/well. Then, the cells were pelleted and suspended in phosphate buffered saline (PBS), washed and supplemented with 0.5% bovine serum albumin (BSA, Sigma) and 10% FCS for an incubation of 10 min on ice (4°C and in the dark) to prevent non-specific antibody binding to free Fc-receptors on the cells. Subsequently, the cells were centrifuged, washed and suspended in the antibody mixtures described above and incubated in the dark for 30 min on ice. Finally, the cells were washed twice with PBS/0.5% BSA and suspended in 1% paraformaldehyde (LUMC Pharmacy). For the regulatory T-cell staining, following the Yellow ARD incubation and blocking step, cells were stained for surface markers as described above, washed twice with PBS/0.5% BSA and subsequently fixated in transcription factor fixation and permeabilized by buffer (BD) and intranuclear stained with the antibodies CD152, FoxP3, Helios and Ki67 (diluted in permeabilization and washing buffer (BD)) for 40–50 min on ice. Cells were finally suspended in 1% paraformaldehyde and assessed within 24 h while keeping them in the dark at 4°C.

### Flow cytometry

Acquisition on the BD Fortessa flow cytometers was performed within 24 h after staining of the cells was finished. Analysis was performed using DIVA software (BD Biosciences, version 6.2).

### PD-1 blocking and stimulation of PBMC in vitro

Thawed autologous monocytes (1–4 × 10^6^ cells/mL) were adhered to the bottom of the wells in a 96-wells plate in X-vivo 15 medium (Lonza) during 2 h incubation at 37°C, 5% CO_2_ in a humidified incubator. The wells were subsequently gently washed to remove non-adherent responder cells (which were centrifuged and stored in a 15 mL tube in IMDM with 10% human AB serum and rested overnight in the incubator) and the adhered monocytes were replenished in 75 µL X-vivo 15 medium with 800 U/mL granulocyte macrophage colony-stimulating factor (GM-CSF) and incubated for 5 h in the incubator. Then, the monocytes were overnight loaded in triplicate wells with 75 µL of the FLU peptide pool (at a concentration of 5 µL/mL) diluted in X-vivo 15 medium. Medium only served as a negative control. The next day, responder cells (50,000–100,000 cells/well) were added as well as the anti-PD-1 antibody Nivolumab (final concentration of 1 µg/mL). After 5 d of incubation, 50 µL supernatant per well was harvested and stored at −20°C for cytokine analysis. The cells were pulsed with [^3^H]-thymidine (PerkinElmer, the Netherlands), and filters were counted using a βplate counter.

### Laboratory environment

Immunomonitoring of patient's PBMCs was performed in the laboratory of the department of Medical Oncology at LUMC that operates under research conditions, following standard operating procedures (SOPs) and using trained personnel. The authors acknowledge the reporting of results from T-cell assays according to the minimal information about T-cell assays (MIATA). Definitions of positive responses were pre-established. This laboratory has been audited both internally and externally, according to the reflection paper for laboratories that perform immunomonitoring.^44^ The laboratory has participated in all proficiency panels of the CIMT Immunoguiding Program (CIP; http://www.cimt.eu/workgroups/cip/) as well as many of the proficiency panels of the USA-based Cancer Immunotherapy Consortium (CIC of the Cancer Research Institute), whose aim is to harmonize reporting and assays used for immunomonitoring and to validate SOPs.

### Statistical interpretation

The repeated measured immune responses of patients were analyzed with a mixed model analysis of variance with fixed factors time, if feasible chemotherapy and chemotherapy by time, random factor subject. Contrasts calculated within the model included: different time points, with or without chemotherapeutic treatment. The fold change in MRM and FLU, absolute shift in lymphoid and myeloid cells, and PD-1 expressing CD4^+^ T cells were analyzed with a repeated measures regression analysis with a compound structure covariance structure and time as repeated factor within each subject. Analysis results per variable are generated with estimates of the difference of the different contrasts and a back transformed estimate of the difference in percentage for log transformed parameters, 95% confidence intervals (in percentage for log-transformed parameters) and least square means (LSM, geometric means for log transformed parameters), and a *p*-value of the contrasts. A *p*-value < 0.05 was considered statistically significant. All calculations and statistical analysis were performed using SAS for Windows V9.4 (SAS Institute, Inc., Cary, NC, USA)

## Supplementary Material

Supplementary_materials.zip
