# Supplementary figures and images for: Impact of (chemo)radiotherapy on immune cell composition and function in cervical cancer patients
Source: Oncoimmunology. 2016 Dec 23;6(2):e1267095. doi: 10.1080/2162402X.2016.1267095 (PMC5353924; doi:10.1080/2162402X.2016.1267095)

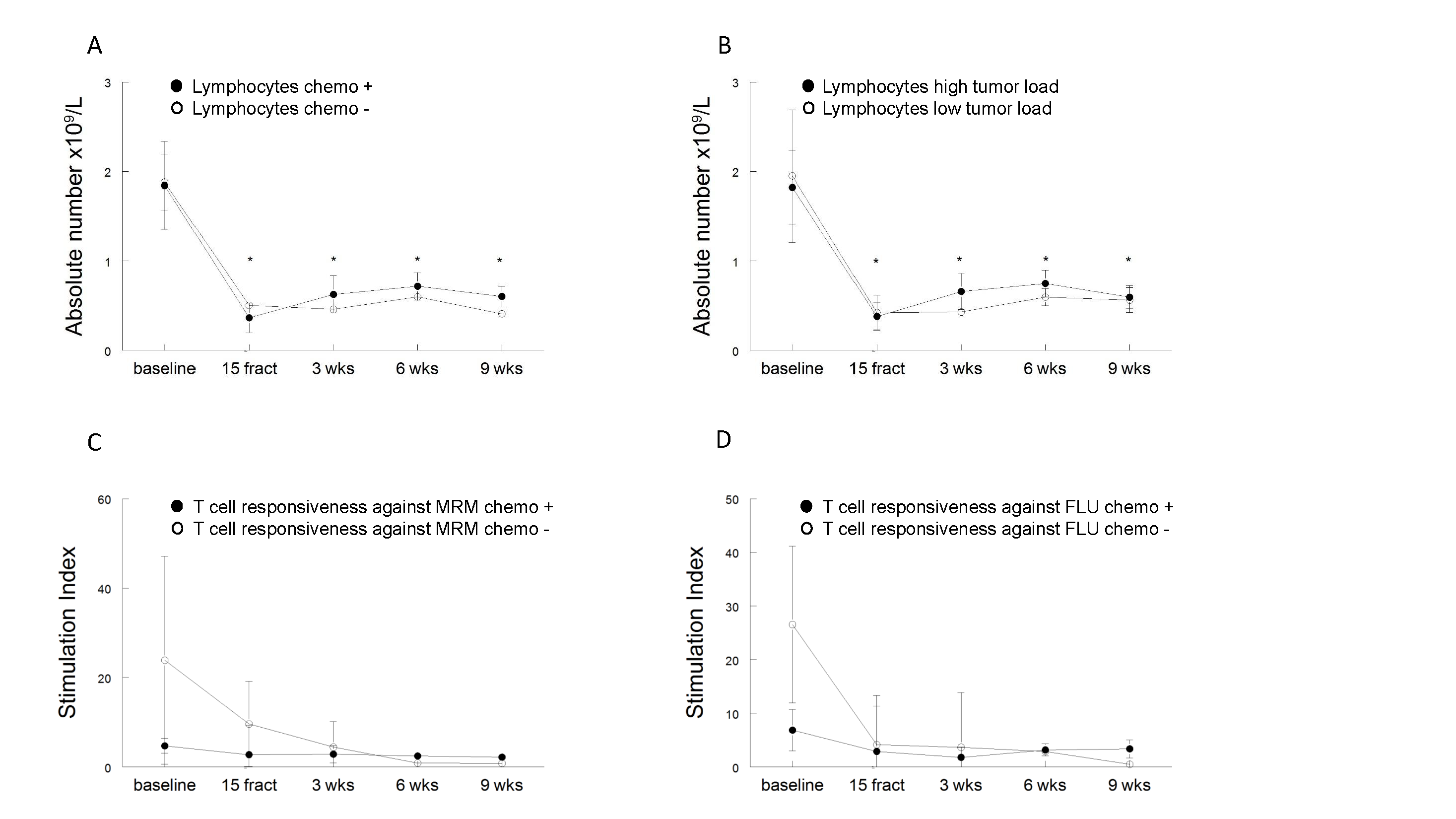

Supplement: Supplementary_materials.zip [file koni-06-02-1267095-s001.zip › Supplemetary Figure_1.tif]

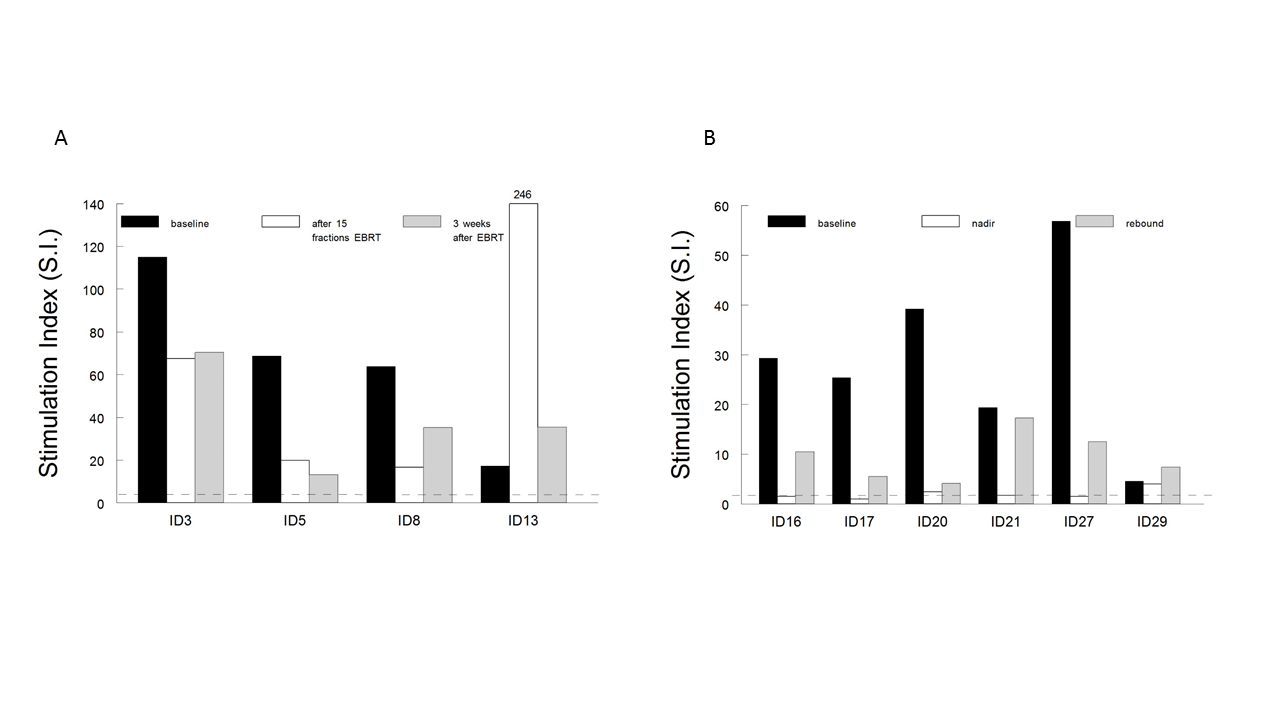

Supplement: Supplementary_materials.zip [file koni-06-02-1267095-s001.zip › Supplemetary Figure_2.tif]

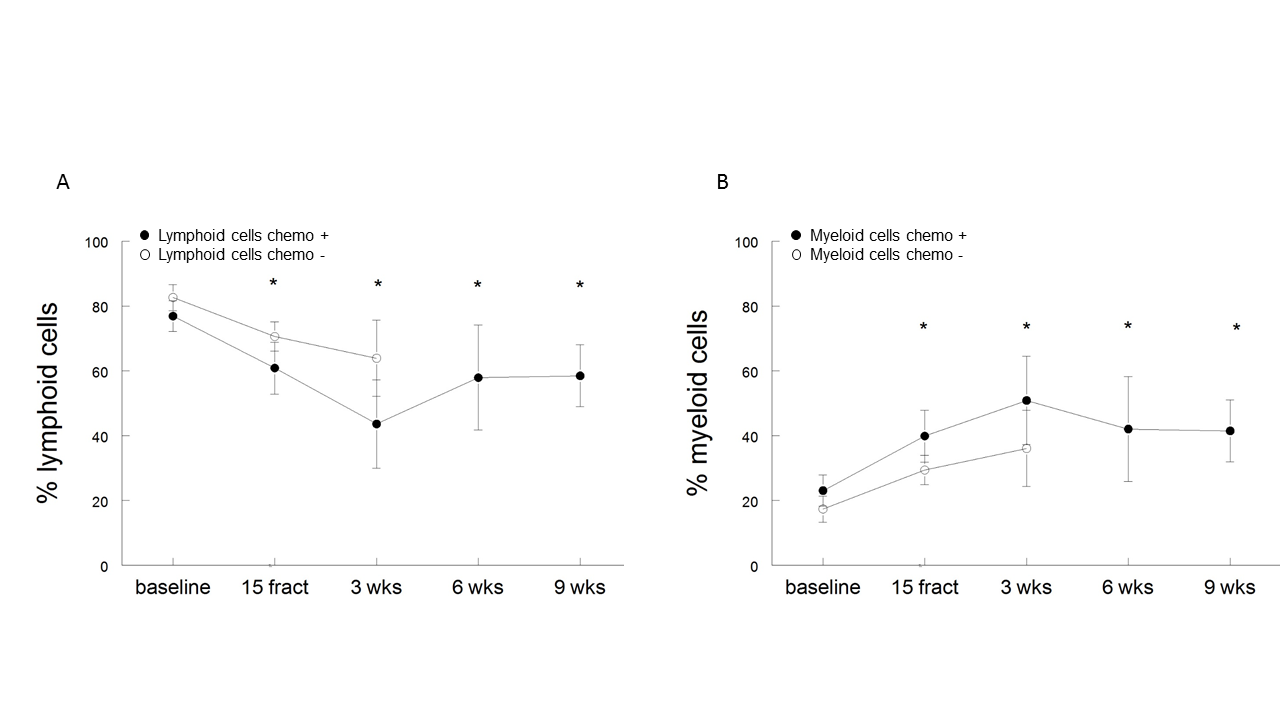

Supplement: Supplementary_materials.zip [file koni-06-02-1267095-s001.zip › Supplemetary Figure_3.tif]

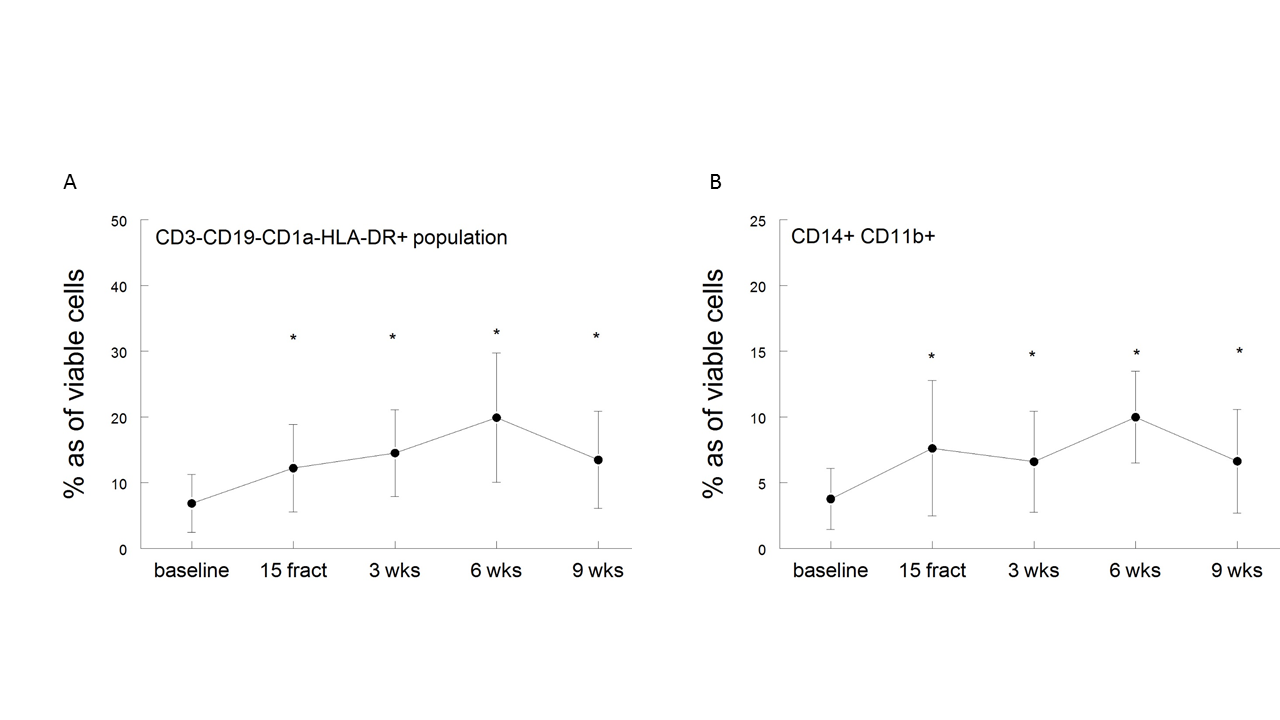

Supplement: Supplementary_materials.zip [file koni-06-02-1267095-s001.zip › Supplemetary Figure_4.tif]

A

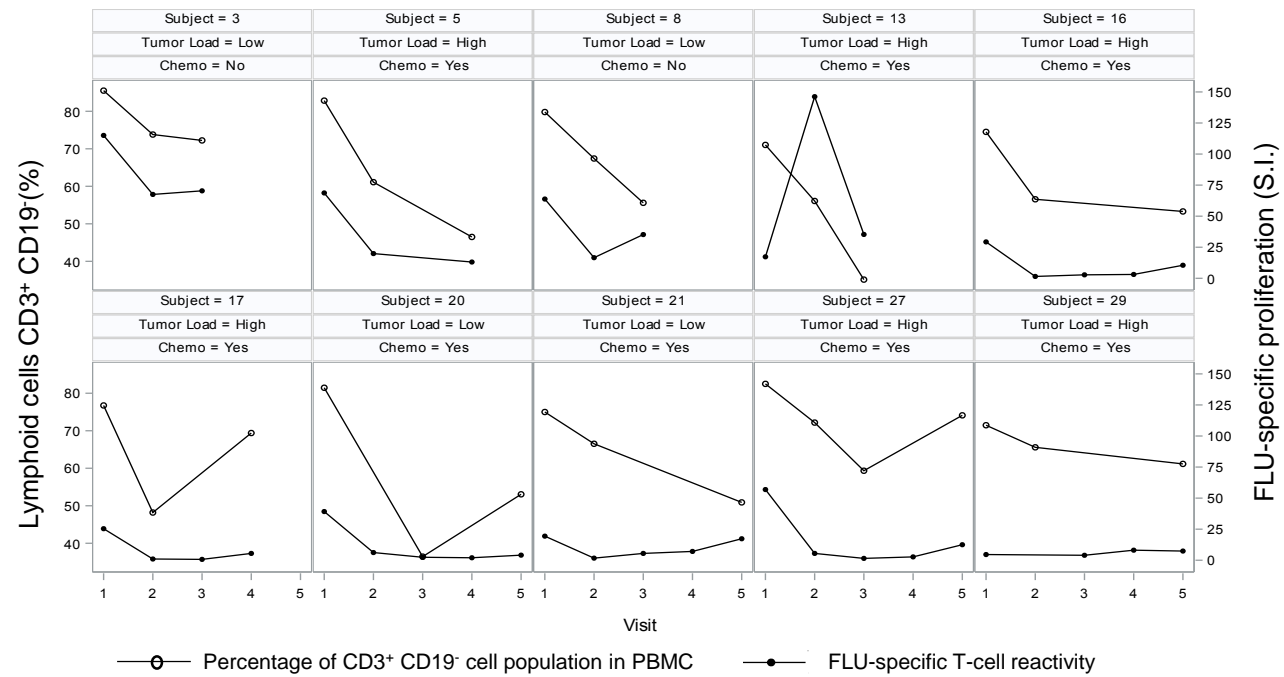

B

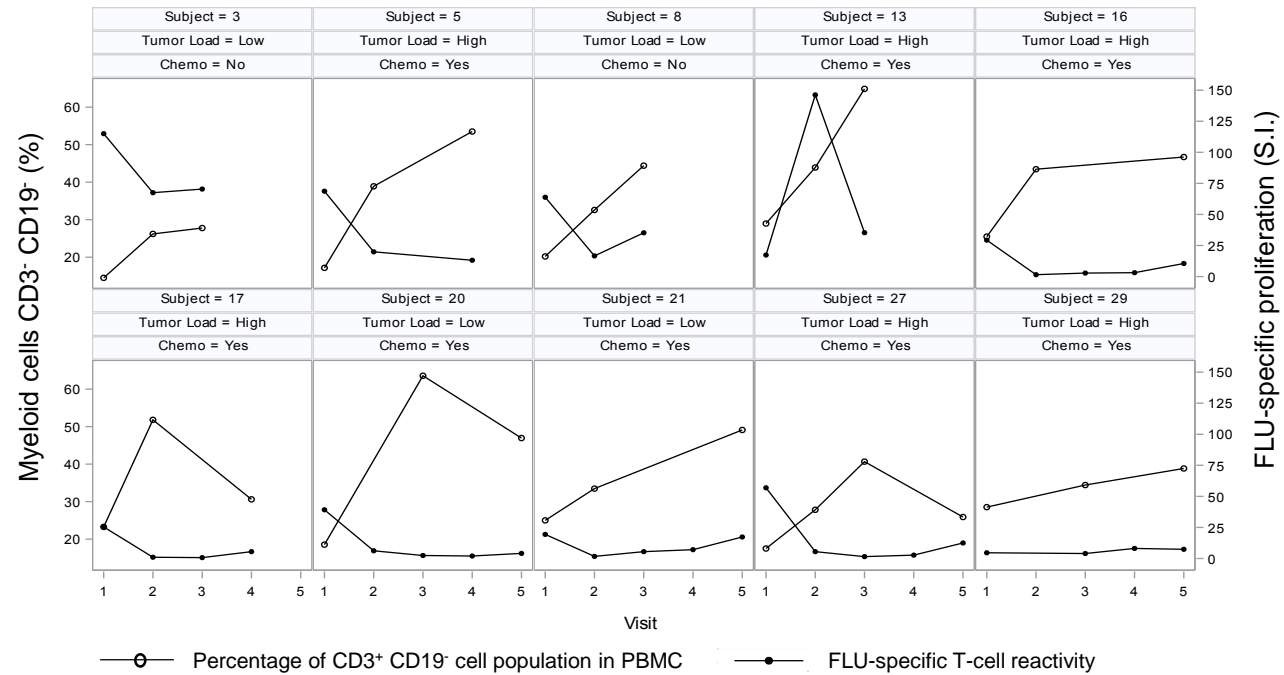

Supplement: Supplementary_materials.zip [file koni-06-02-1267095-s001.zip › Supplemetary Figure_5.pdf]

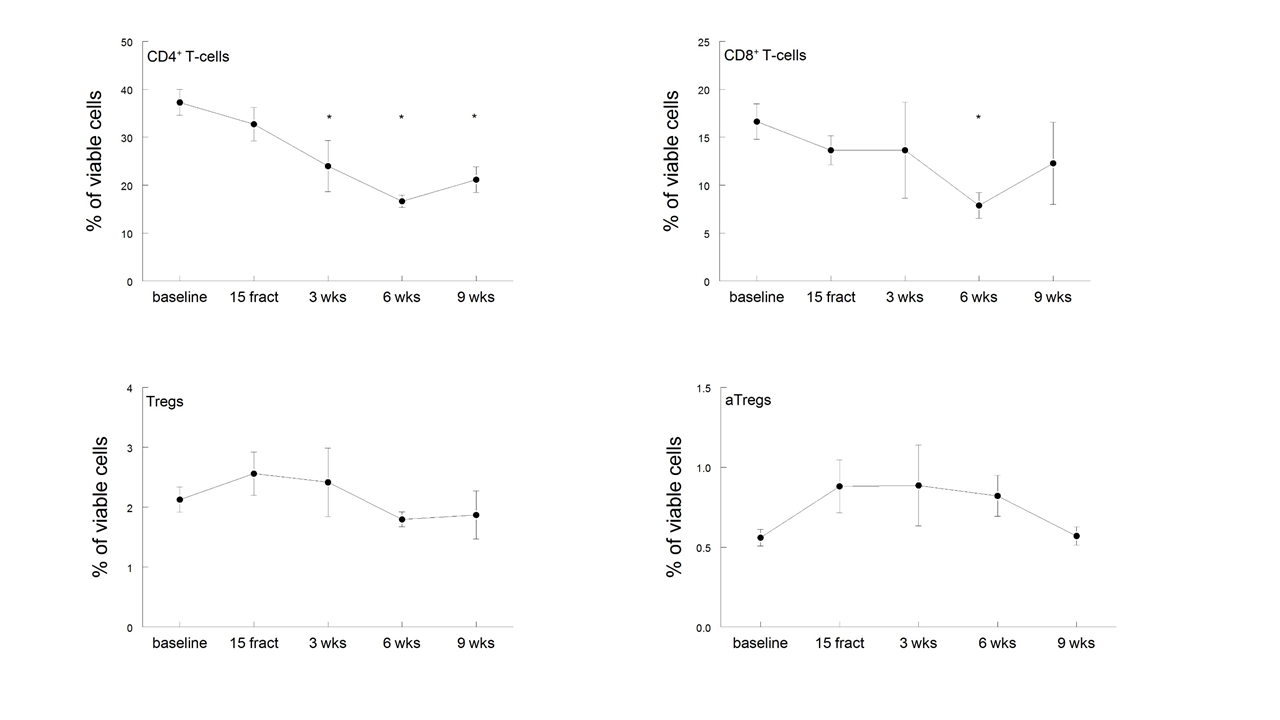

Supplement: Supplementary_materials.zip [file koni-06-02-1267095-s001.zip › Supplemetary Figure_6.tif]

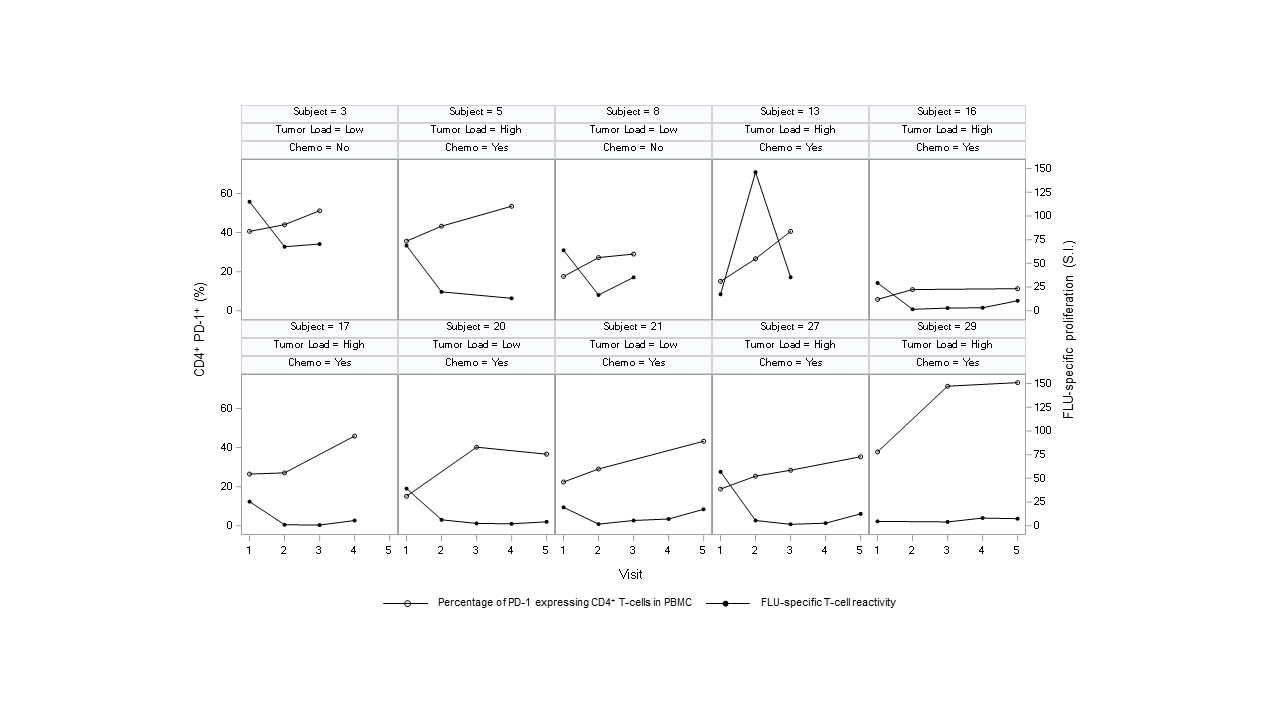

Supplement: Supplementary_materials.zip [file koni-06-02-1267095-s001.zip › Supplemetary Figure_7.tif]
