# Supplementary material for: Impact of (chemo)radiotherapy on immune cell composition and function in cervical cancer patients
Source: Oncoimmunology. 2016 Dec 23;6(2):e1267095. doi: 10.1080/2162402X.2016.1267095 (PMC5353924; doi:10.1080/2162402X.2016.1267095)
Supplement: Supplementary_materials.zip [file koni-06-02-1267095-s001.zip › Supplementary Legend.doc]

**Supplementary figure 1.** The effect of therapy on circulating lymphocytes as measured in whole blood, comparing patients receiving EBRT only or EBRT in combination with chemotherapy (A), and comparing patients receiving (chemo-)radiation with a high or low tumor load (B). The effect on T-cell responsiveness against MRM and FLU with a lymphocyte stimulation test (LST), is also compared between patients receiving EBRT alone or in combination with concurrent chemotherapy (C, D).

Time course of changes before, during and 3, 6 and 9 weeks after radiotherapy. The suppressing effect of radiotherapy on peripheral lymphocytes and T-cell responses was similar for patients treated with radiotherapy alone or with concomitant chemotherapy (A, C, D) and regardless of whether tumor-load was high or low (B). Data are expressed as means ± SD ; * p < 0.05 compared to baseline.

Abbreviations: wks = weeks; fract = fractions

**Supplementary figure 2.** The effect of EBRT on circulating T-cell responses in individual patients as measured in the LST against FLU. A. Dynamics for 4 patients with a strong T-cell response as indicated by the stimulation index (SI), at baseline (black bars), during (white bars) or after (gray bars) EBRT treatment. B. T-cell response dynamics for 6 patients showing a rebound of T cell responses after completion of EBRT. Baseline is indicated by black bars. Nadir (white bars) indicates midway or close after last EBRT treatment. Rebound (gray bars) is at 6 to 9 weeks after last EBRT.

--- line represents S.I. value of 3, a positive T-cell response is defined as a S.I. of 3 or higher.

Abbreviations: EBRT = external beam radiation therapy; S.I. = Stimulation Index; LST = Lymphocyte Stimulation Test

**Supplementary Figure 3.** The treatment effect on (**A.**) circulating lymphoid cells (CD3^+^CD19^-^) and (**B.**) myeloid cells (CD3^-^CD19^-^) of viable cells as measured by flow cytometry, compared between patients receiving EBRT alone or in combination with concurrent chemotherapy. Both radiotherapy alone (2 patients) and radiotherapy with concurrent cisplatin chemotherapy (8 patients) altered the relative frequencies of circulating myeloid and lymphoid cells. Longer follow-up (6 and 9 weeks after completion of EBRT) is missing for the 2 patients treated with EBRT alone.

Percentage are expressed as mean ± SD. *p<0.05 with respect to baseline. Abbreviations: wks = weeks; fract = fraction; chemo = concurrent chemotherapy with cisplatin.

**Supplementary Figure 4.** The effect of (chemo)radiotherapy on the percentage of circulating CD3^-^CD19^-^CD1a^-^HLA^-^DR^+^ population (A) and on the subpopulation of monocytes expressing both CD14 and CD11b (B). Data is expressed as percentage of viable cells. *p<0.05 with respect to baseline.

**Supplementary Figure 5**. T-cell responsiveness against FLU within LST, presented for 10 individual patients showing high responsiveness at baseline, severe drop upon (chemo)radiotherapy (except for subject 13) and slight rebound 6-9 weeks after completion of EBRT in 6 cases. The strong decrease in T-cell responsiveness occurred concurrently with a decrease in percentage of circulating CD3^+^CD19^-^ lymphoid cells (A), and an increase in percentage of circulating CD3^-^CD19^-^ myeloid cells (B) in 9 of the 10 patients. Visit 1: baseline. Visit 2: after 15 fractions of EBRT. Visit 3: 3 weeks after EBRT. Visit 4: 6 weeks after EBRT. Visit 5: 9 weeks after EBRT.

**Supplementary Figure 6.** The effect of radiotherapy on the percentage of circulating CD4^+^ T cells, CD8^+^ T cells, CD4^+^CD25^+^CD127^-^Foxp3^+^ regulatory T-cells (Treg), and CD4^+^CD25^+^CD127^-^Foxp3^+^CD45RA^-^ activated Tregs (aTreg). Data is expressed as percentage of viable cells and presented using mean values ± SEM. *p<0.05 with respect to baseline.

**Supplementary Figure 7.** Individual plots, showing T-cell responsiveness against FLU within LST. At baseline, positive responsiveness (S.I.≥3) is seen in all patients. A severe drop in T-cell responsiveness occurs upon EBRT (except for subject 13) and slight rebound 6-9 weeks after completion of EBRT in 6 cases. Except for ID 13, the strong decrease in T-cell responsiveness occurred concurrently with an increase in PD-1 expression on CD4^+^ T-cells.

Visit 1: baseline. Visit 2: after 15 fractions of EBRT. Visit 3: 3 weeks after EBRT. Visit 4: 6 weeks after EBRT. Visit 5: 9 weeks after EBRT.

| **Patient ID** | **Age** | **Treatment** | **Dosages of ChTh, EBRT and BT** | **Treatment for** | **Tumor burden** |
| --- | --- | --- | --- | --- | --- |
| 1 | 31 | EBRT + ChTh + BT | 23x2Gy EBRT; 6xCis; 4xBT | primary disease | high |
| 2 | 54 | EBRT + BT | 23x2Gy EBRT; 3xBT | recurrent disease | high |
| 3 | 41 | EBRT | 23x2Gy EBRT | adjuvant | low |
| 4 | 40 | EBRT | 20x2Gy EBRT | adjuvant | low |
| 5 | 56 | EBRT + ChTh + BT | 27x1.8Gy EBRT; 6xCis; 3xBT | recurrent disease | high |
| 6 | 67 | EBRT + ChTh | 30x2Gy EBRT; 6xCis | adjuvant | low |
| 7 | 28 | EBRT + ChTh + BT | 23x2Gy EBRT; 5xCis; 3xBT | primary disease | high |
| 8 | 19 | EBRT | 30x2Gy EBRT | adjuvant | low |
| 9 | 82 | EBRT + BT | 23x2Gy EBRT; 2xBT | adjuvant | low |
| 10 | 26 | EBRT + ChTh + BT | 28x2Gy EBRT; 3xCis; 3xBT | primary disease | high |
| 11 | 49 | EBRT + ChTh + BT | 28x2Gy EBRT; 6xCis; 3xBT | primary disease | high |
| 12 | 36 | EBRT + ChTh | 30x2Gy EBRT; 6xCis | recurrent disease | high |
| 13 | 53 | EBRT + ChTh + BT | 27x1.8Gy EBRT; 6xCis; 3xBT | primary disease | high |
| 14 | 62 | EBRT + ChTh + BT | 25x1.8Gy EBRT; 3xCis; 3xBT | primary disease | high |
| 15 | 35 | EBRT + ChTh + BT | 23x2Gy EBRT; 6xCis; 3xBT | primary disease | high |
| 16 | 68 | EBRT + ChTh + BT | 23x2Gy EBRT; 6xCis; 3xBT | primary disease | high |
| 17 | 50 | EBRT + ChTh | 33x2Gy EBRT; 6xCis | recurrent disease | high |
| 18 | 55 | EBRT + ChTh + BT | 25x1.8Gy EBRT; 5xCis; 3xBT | primary disease | high |
| 19 | 33 | EBRT | 23x2Gy EBRT | adjuvant | low |
| 20 | 45 | EBRT + ChTh | 27x1.8Gy EBRT; 6xCis | adjuvant | low |
| 21 | 35 | EBRT + ChTh | 23x2Gy EBRT; 5xCis | adjuvant | low |
| 22 | 69 | EBRT + ChTh + BT | 25x1.8Gy EBRT; 5xCis; 3xBT | primary disease | high |
| 23 | 69 | EBRT + ChTh + BT | 27x1.8Gy EBRT; 5-FU; 3xBT | primary disease | high |
| 24 | 51 | EBRT + ChTh + BT | 23x2Gy EBRT; 6xCis; 3xBT | primary disease | high |
| 25 | 53 | EBRT + ChTh + BT | 23x2Gy EBRT; 6xCis; 3xBT | primary disease | high |
| 26 | 79 | EBRT + HT | 23x2Gy EBRT; 4xHT | primary disease | high |
| 27 | 51 | EBRT + ChTh + BT | 23x2Gy EBRT; 6xCis; 3xBT | primary disease | high |
| 28 | 54 | EBRT + ChTh + BT | 27x1.8Gy EBRT; 5xCis; 3xBT | primary disease | high |
| 29 | 49 | EBRT + ChTh + BT | 27x1.8Gy EBRT; 6xCis; 3xBT | primary disease | high |
| 30 | 28 | EBRT + ChTh + BT | 30x1.8Gy EBRT; 5xCis; 3xBT | primary disease | high |

**Supplementary table I.** Detailed patient treatment characteristics. Age is expressed in years. Tumor burden was identified as high or low, with high tumor burden in case of primary or recurrence therapy; and low tumor burden in case of adjuvant, post-operative therapy. All treatments were highly dosed: chemotherapy with cisplatin at a dose of 40 mg/square meter, in 5-6 cycles ; radiotherapy in 23 to 30 fractions of 1.8 or 2 Gray (Gy). Abbreviations: BT = brachytherapy ; Cis = cisplatin; ChTh = chemotherapy; EBRT = external beam radiation therapy ; HT = hyperthermia; 5-FU = 5 Fluorouracil.
